# Supplementary material for: DMRforPairs: identifying Differentially Methylated Regions between unique samples using array based methylation profiles
Source: BMC Bioinformatics. 2014 May 15;15:141. doi: 10.1186/1471-2105-15-141 (PMC4046028; doi:10.1186/1471-2105-15-141)
Supplement: Additional file 2 — DMRforPairs output for the comparison of A431-MCF7 and NA17018-NA17105. Please start from the HTML files in each folder. Available via the BMC Bioinformatics website. [file 1471-2105-15-141-S2.zip › 1394847754114233_MOESM2_ESM/A431_MCF7/figures/15056.pdf]

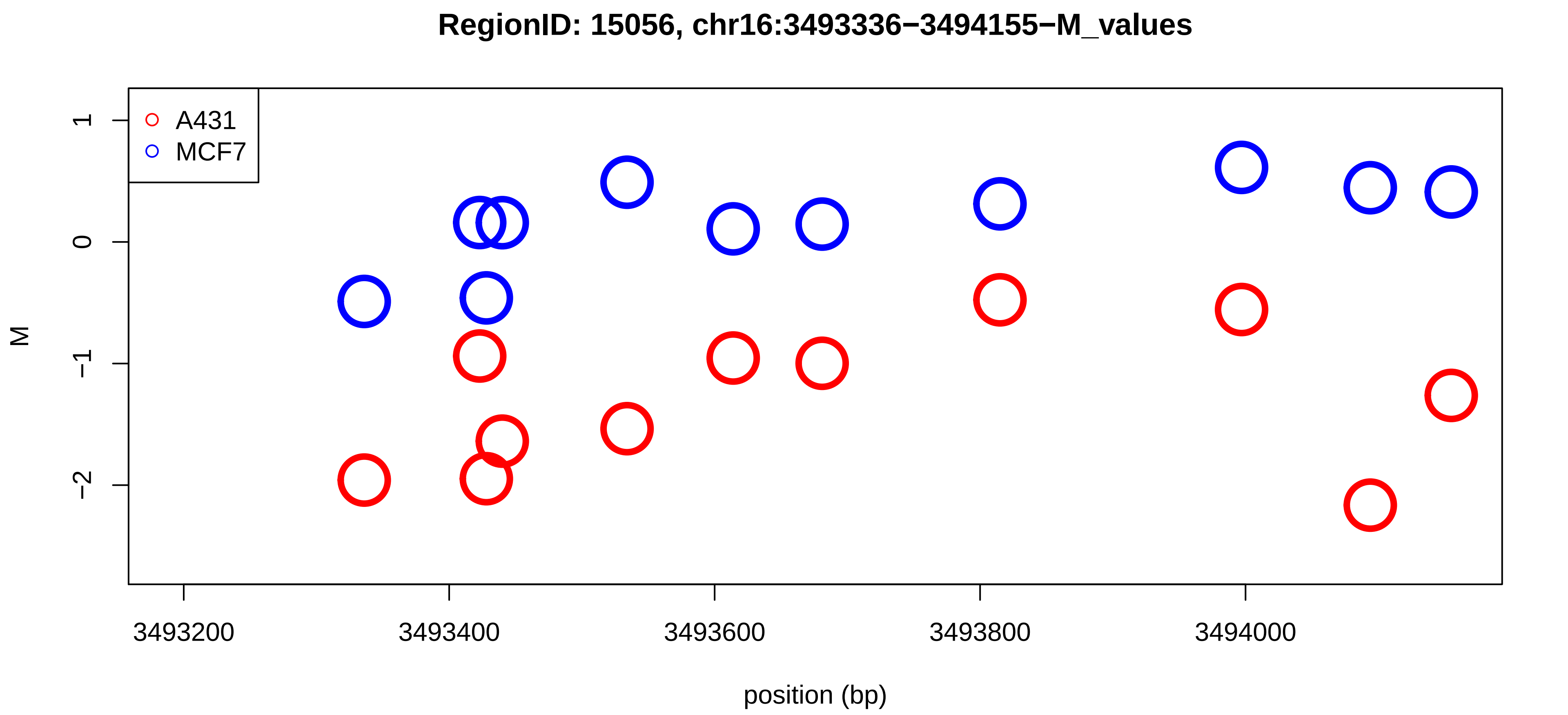

RegionID: 15056, chr16:3493336−3494155−M_values
A431
MCF7
M
1
0
−1
−2
3493200
3493400
3493600
3493800
3494000
position (bp)


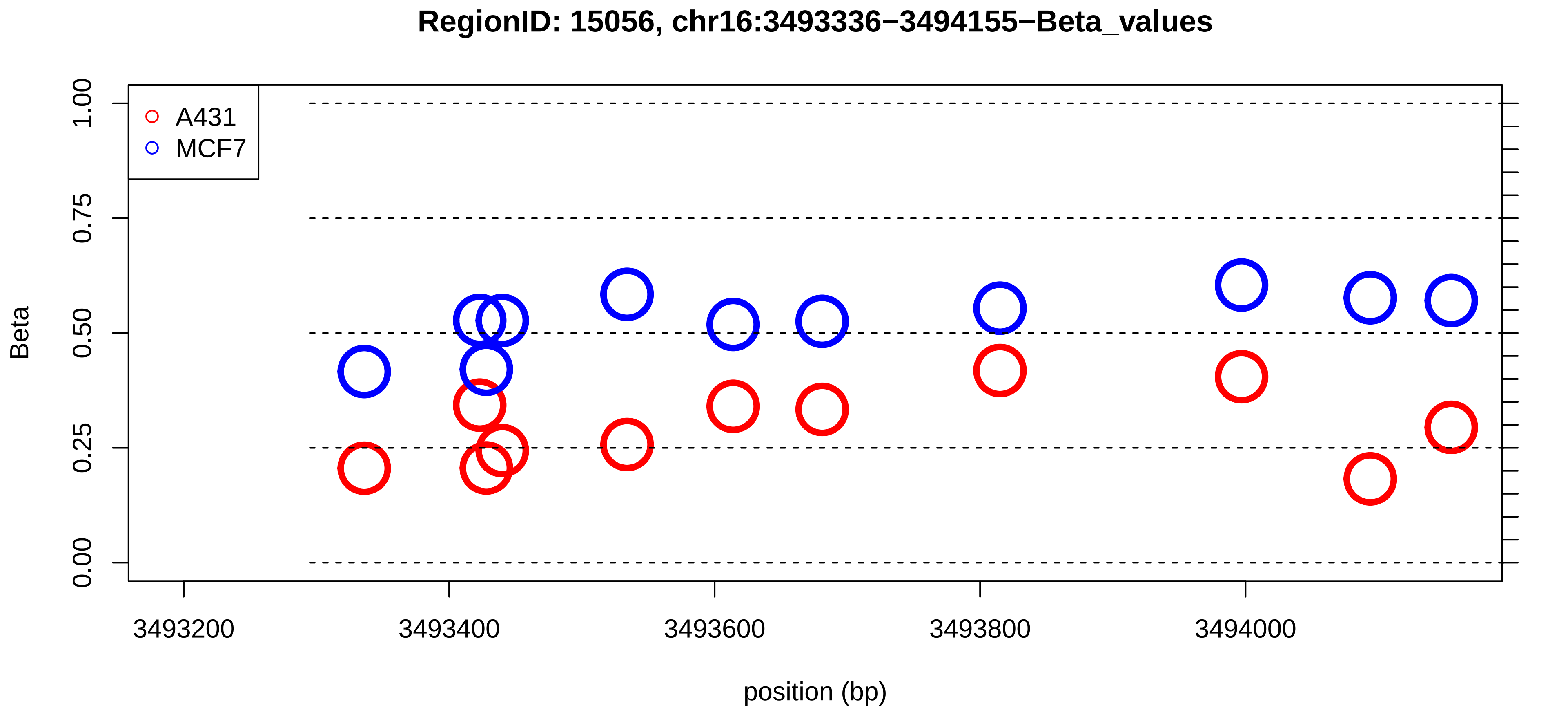

RegionID: 15056, chr16:3493336−3494155−Beta_values
A431
MCF7
Beta
1.00
0.75
0.50
0.25
0.00
3493200
3493400
3493600
3493800
3494000
position (bp)

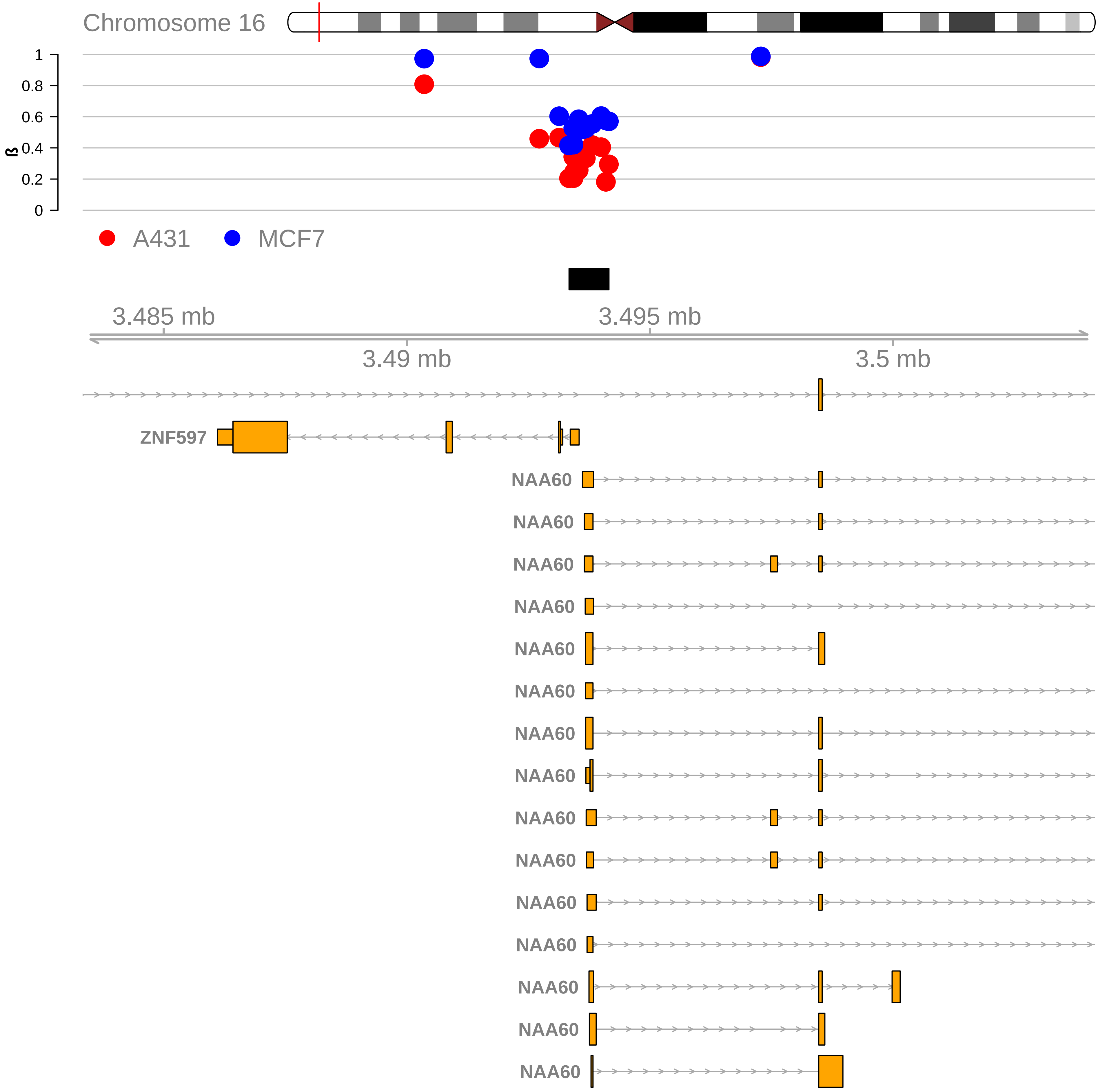

Chromosome 16
1
0.8
0.6
ß 0.4
0.2
0
A431
MCF7
3.485 mb
3.495 mb
3.49 mb
3.5 mb
ZNF597
NAA60
NAA60
NAA60
NAA60
NAA60
NAA60
NAA60
NAA60
NAA60
NAA60
NAA60
NAA60
NAA60
NAA60
NAA60
